# Typhoid

**Published:** 1915-09

**Authors:** 


**A Monthly Review of Medicine and Surgery**

EDITOR AND PUBLISHER, DR. A. L. BENEDICT, 228 Summer St., corner of Elmwood Ave., Buffalo, (Address for all communications. Please make personal and telephone calls before 1 P. M.)

**Third, in age, on the western continent. Independent, but supports professional organization. News limited mainly to Western N. Y. and Penn.**

**Subscription $2.00 a year; $3.00 for two years in advance. Changes and errors in address or failure or duplication of delivery, should be reported immediately.**

Yearly Volume 71 SEPTEMBER, 1915 No. 2

### Typhoid.

With possible rare exceptions, typhoid is a human disease. With every allowance for contact infection which is usually limited to persons closely associated with an actual case or a "carrier," for conveyance by flies, etc., and for infection of milk, garden vegetables and other foods, the great majority of cases are due to infected water, and to water as a liquid, ice conveyance being slight. Without typhoid and cholera Asiatica, almost any degree of filthiness in the admission of dejecta to water supplies could be tolerated without actual danger since these are the only two essentially hydrophoric diseases—with the possible exception of certain local diseases not familiar to European and American practitioners.

Any reasonably thorough method of preventing infection through water—as by boiling, sand filtration, or even chemic treatment—rapidly reduces the incidence of typhoid to somewhere between 40 and ten cases a year per hundred thousand population, the lower figure representing what may be regarded under present conditions as an inevitable incidence. Even a higher incidence does not necessarily indicate inefficient purification of the water supply. In Buffalo, for example, the seasonal incidence and study of place of occupation, circumstances of infection, etc., show that at least half of the typhoid cases originate outside the city.

On the other hand, even a slight proportionate incidence of typhoid in a community, with precautions against local spread of infection, magnifies the prevalence of the disease in any community to whose water supply the former's sewage is tributary. The doctor or nurse who does not disinfect typhoid stools and urine—the latter containing bacilli in about 25% of samples analysed and probably in nearly 100% of the aggregate urines for the course of the disease—is close to manslaughter. But a great many are heedless, many cases go unrecognized for several days, even for weeks. So far as we know, NO BOARD OF HEALTH, ANYWHERE, MAKES SYSTEMATIC BACTERIOLOGIC EXAMINATIONS OF TYPHOID FAECES TO DETERMINE WHEN A PATIENT SHOULD BE DISCHARGED FROM QUARANTINE. It is estimated that 3-5% of typhoid cases become carriers and as the discharges have been shown to be infectious many years later, it is obvious that, in a few years, the carriers in any community represent more potential infection than the maximum of actual acute cases.

We have purposely used the term **community** in referring to the magnification of typhoid by passage through human cases. The community may be a family with a privy draining into a brook or, along a seam of rock into a neighbor's well; it may be a large city discharging sewage into a river. What we want to emphasize is that the danger of infection can not be gauged by the actual or relative number of cases of typhoid in the former. In fact, it is the one or two cases in the country, the dozen or two in the village that represent enormous incidence rates, not the epidemics of hundreds or thousands of cases in large cities. But, for some years to come, we shall have the puzzling problem of the carrier—puzzling diagnostically legally and therapeutically. Moreover, relative volume of sewage and potable streams, rapidity of current and other factors influencing sedimentation, oxidation, etc., are probably of more importance than number of cases.

